# The symbiotic relationship between *Caenorhabditis elegans* and members of its microbiome contributes to worm fitness and lifespan extension

**DOI:** 10.1186/s12864-021-07695-y

**Published:** 2021-05-19

**Authors:** Orçun Haçariz, Charles Viau, Farial Karimian, Jianguo Xia

**Affiliations:** 1 Institute of Parasitology, McGill University, Montreal, Quebec Canada; 2 Department of Animal Science, McGill University, Montreal, Quebec Canada

**Keywords:** RNAseq, Nanopore sequencing, *C. elegans*, Host-microbiome interaction, Cellular detoxification, Signaling pathways, Lifespan

## Abstract

**Background:**

A healthy microbiome influences host physiology through a mutualistic relationship, which can be important for the host to cope with cellular stress by promoting fitness and survival. The mammalian microbiome is highly complex and attributing host phenotypes to a specific member of the microbiome can be difficult. The model organism *Caenorhabditis elegans* and its native microbiome, discovered recently, can serve as a more tractable, experimental model system to study host-microbiome interactions. In this study, we investigated whether certain members of *C. elegans* native microbiome would offer a benefit to their host and putative molecular mechanisms using a combination of phenotype screening, omics profiling and functional validation.

**Results:**

A total of 16 members of *C. elegans* microbiome were screened under chemically-induced toxicity. Worms grown with *Chryseobacterium* sp. CHNTR56 MYb120 or *Comamonas* sp. 12022 MYb131, were most resistant to oxidative chemical stress (SiO_2_ nanoparticles and juglone), as measured by progeny output. Further investigation showed that *Chryseobacterium* sp. CHNTR56 positively influenced the worm’s lifespan, whereas the combination of both isolates had a synergistic effect. RNAseq analysis of young adult worms, grown with either isolate, revealed the enrichment of cellular detoxification mechanisms (glutathione metabolism, drug metabolism and metabolism of xenobiotics) and signaling pathways (TGF-beta and Wnt signaling pathways). Upregulation of cysteine synthases (*cysl* genes) in the worms, associated with glutathione metabolism, was also observed. Nanopore sequencing uncovered that the genomes of the two isolates have evolved to favor the specific route of the de novo synthesis pathway of vitamin B6 (cofactor of cysl enzymes) through *serC* or *pdxA2* homologs. Finally, co-culture with vitamin B6 extended worm lifespan.

**Conclusions:**

In summary, our study indicates that certain colonizing members of *C. elegans* have genomic diversity in vitamin B6 synthesis and promote host fitness and lifespan extension. The regulation of host cellular detoxification genes (i.e. *gst*) along with *cysl* genes at the transcriptome level and the bacterium-specific vitamin B6 synthesis mechanism at the genome level are in an agreement with enhanced host glutathione-based cellular detoxification due to this interspecies relationship. *C. elegans* is therefore a promising alternative model to study host-microbiome interactions in host fitness and lifespan.

**Supplementary Information:**

The online version contains supplementary material available at 10.1186/s12864-021-07695-y.

## Background

Through evolution, symbiotic microorganisms are found to be in a wide variety of relationships with their host, which can be categorised as parasitic, commensal or mutualistic. The normal mammalian microbiome (defined as the totality of microbe species found in or on the host) has evolved to be mainly mutualistic, with significant effects on host development, immunity and metabolism [1–4]. Interruptions in the balance of such host-microbiome relationships, known as dysbiosis, can lead to diseases [5]. Understanding such complex interactions is critical to the development of rational microbial therapies. However, elucidating causal relationships between members of the microbiome and mammalian hosts is difficult due to diverse factors such as host genetics and environmental variability [6]. Mice are commonly used to study host-microbiome interactions based on their similarities to humans in terms of genetics, immune system, as well as the anatomy and physiology of the digestive tract [7]. However, the mouse model has limited throughput and complex genetic interactions with its microbiome [8]. In addition, the cost associated with microbiome studies on mammals can be exorbitant. Simplified, less costly alternative models are often desirable [7].

The use of alternative models to study host-microbiome interactions has gained traction in recent years. For example, *Drosophila melanogaster*, the fruit fly, with its simple microbiome and its tractability and high-throughput capability, is a well-established model to study the effects of the microbiome on the host, including mate selection [9–11]. Another model organism that has become attractive in studying the microbiome is the nematode bacterivore *Caenorhabditis elegans*. The bacteria species comprising the native microbiome of this model organism were characterized by several research groups in 2016 [12–14].

The native microbiome of *C. elegans* mainly consists of four bacteria phyla including *Bacteroidetes*, *Actinobacteria*, *Firmicutes* and *Proteobacteria* [12, 15]. These four phyla are also present in human gut microbiome [6]. Since then, several studies have highlighted the impact of the *C. elegans* microbiome on the physiology of the worm. For example, Cassidy et al. [16] investigated the effects of *Ochrobactrum* isolates on *C. elegans* and demonstrated that the levels of the worms’ protein expression (lipase, proteases and glutathione metabolism) were increased and the levels of the worm’s proteins related to both degradation and biosynthesis of amino acids were decreased. Yang et al. [17] showed that *Ochrobactrum* isolates modulated *C. elegans* physiology through metabolism of specific amino acids, fatty acids, and also folate biosynthesis. However, the biological influence of the vast majority of the native microbiome members of *C. elegans* has not yet been investigated.

As *C. elegans* is a bacterivore, phenotypes of worms grown with a single bacterial isolate (i.e. monoxenic cultures) can be screened and studied in terms of fitness in response to chemical perturbations. A chemical perturbation usually causes cellular oxidative stress, which has effects on host fitness, such as reproduction (progeny output) [18–21]. To establish this in experimental studies, various chemicals including SiO_2_ nanoparticles and juglone, can be used. SiO_2_ nanoparticles and juglone, are classified as metal oxide nanoparticles and a naphthoquinone, respectively [22, 23]). Both SiO_2_ nanoparticles and juglone cause oxidative stress by generating reactive oxygen species (ROS) which can react with nucleic acids, proteins and lipids, and damage the cell [23, 24].

The native bacteria, which are fed to the worm, can colonize the worm’s inner surfaces (the most likely sites being the pharynx and intestine). Colonization by the members of the microbiome in *C. elegans* can be confirmed by Fluorescence In Situ Hybridization (FISH) or by destruction of antibiotic-treated *C. elegans* and visual inspection of colonies after plating the homogenate on plates [12, 25]. After colonization, bacteria can potentially modulate of the effect of chemical perturbations by interacting with the host.

We hypothesized that microbiome members of *C. elegans* would have a beneficial effect on the worm under oxidative stress conditions by using SiO_2_ and juglone, of which toxicity is known to be mediated through cellular oxidative stress [19, 20]. We first evaluated the 16 known members of the *C. elegans* native microbiome in SiO_2_ nanoparticle toxicity screening, by testing the young adult worms’ response in dealing with toxicity as measured by progeny output. Based on the results, we selected two bacterial isolates (*Chryseobacterium* sp. CHNTR56 MYb120 and *Comamonas* sp. 12022 MYb131 and investigated their effects on the lifespan of the host worms. To gain insights of the potential molecular mechanisms, we further performed a comprehensive RNAseq on the worm hosts and whole genome sequencing on the bacterial isolates. Finally, we validated the effects of the constant supply of an essential vitamin, vitamin B6, hypothesized to be important in the relationship between *C. elegans* and its colonizing native bacterial isolates, on worm lifespan, as suggested by the omics data.

## Results

### Some native bacterial isolates enhance the Worm’s capacity in dealing with toxicity based on increased progeny production

**Fig. 1:**
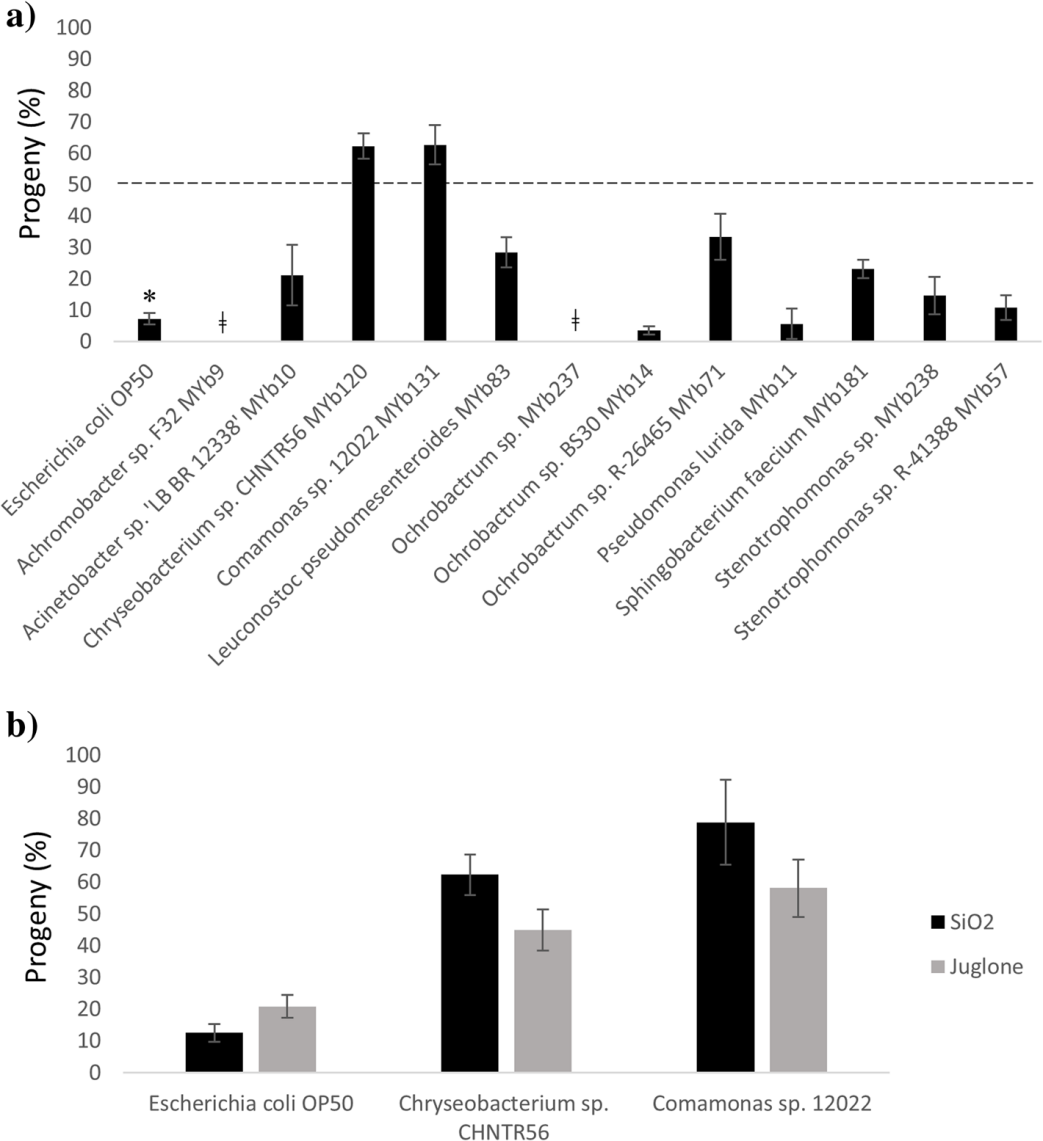
Responses to toxicity as measured by progeny output. **a**. *C. elegans* grown with *Chryseobacterium* sp. CHNTR56 MYb120 or *Comamonas* sp. 12022 MYb131 provided a progeny output (%) greater than 50% (compared to untreated control) under SiO2 toxicity (50 μg/ml of SiO_2_) in the initial screening. **b.** The progeny output for the worms grown with *Chryseobacterium* sp. CHNTR56 MYb120 or *Comamonas* sp. 12022 MYb131, compared to the worms grown with *E. coli* OP50, in the presence of SiO_2_ or juglone (50 μM) *: The progeny output for *E. coli* OP50 is the mean value calculated from each plate used (value for standard error of mean was negligible, 3.61%). ǂ: No progeny detectable under toxicity

As microbiome members are implicated in host fitness, we tested their effects on *C. elegans* reproduction (progeny output) under stress conditions by feeding the worm the corresponding bacterial diet. Initially, L1 *C. elegans* larvae were incubated with each of 16 native bacterial isolates (Additional file 1) or *E. coli* OP50 on NGM plates. Several bacteria (*Arthrobacter aurescens* MYb27, *Microbacterium oxydans* MYb45, *Rhodococcus erythropolis* PR4 MYb53 and *Bacillus* sp. SG20 MYb56) could not be included in the analysis as the larva number and/or size of the worms grown with these bacteria was not sufficient. L4-young adult *C. elegans* were then screened for progeny output in a liquid-based assay in the presence of SiO_2_ nanoparticles and the microbiome members. The isolates with better maintained progeny output (> 50% of the control value) (Fig. 1a) were further tested with SiO_2_ and juglone, in triplicate. Worms grown with *Chryseobacterium* sp. CHNTR56 MYb120 or *Comamonas* sp. 12022 MYb131 showed significantly higher ratio in progeny output under SiO_2_ or juglone toxicity, compared to the worms grown with *E. coli* OP50 (*P* < 0.0001) (Fig. 1b).

Total progeny production of the worms grown with *E. coli* OP50 was higher compared to that of the worms grown with the native bacterial isolates, and the progeny production rate between the worms grown with these isolates was mainly similar in the control wells (in the absence of experimentally induced toxicity). Worms grown with *E. coli* OP50 as well as with many of the other bacterial isolates did not show the beneficial effect against toxicity as worms grown with *Chryseobacterium* sp. CHNTR56 MYb120 or *Comamonas* sp. 12022 MYb131 (Fig. 1a). In terms of ratio of progeny production, *Chryseobacterium* and *Comamonas* fed worms far exceeded the ratio for *E. coli* OP50 fed worms (Fig. 1a, b). Overall, these findings suggested that these members of the worm’s microbiome, when fed to the worm, provided a beneficial effect against toxic compounds, observed by the improvement of host fitness (i.e. reproduction) under stress conditions.

### The native bacterial isolates colonize the worm host and extend lifespan

The colonization assay supported that microbiome members interact differently with *C. elegans* compared to *E. coli* OP50. According to recent reports, native microbiome members colonize *C. elegans* more efficiently than non-native bacteria, such as *E. coli* OP50 [12, 14, 17]. By plating on TSB plates with no antibiotics, we determined that the tested native bacteria including *Chryseobacterium* sp*.* CHNTR56 MYb120 or *Comamonas* sp. 12022 MYb131, were still culturable from the worms with no food after a 24-h period (incubated on NGM plates containing effective antibiotics against the native bacterial isolates), indicating that these bacteria have colonized the worm host. This assay was further supported by the absence or negligible number of colonies for the non-native and non-colonizing bacterium, *E. coli* OP50 (Additional file 2, Fig. S1). In Additional file 2, Fig. S1, the number of *E. coli* OP50 colonies (as seen by the colony forming units) recovered is much lesser compared to the number of native bacteria colonies recovered from the worms, indicating that these native bacteria colonize inside the worm. The most likely sites of colonization are the worm intestine and pharynx, as the worms’ outside surface (i.e. cuticle) was sterilized by antibiotic treatment. The colonization assay suggested that *C. elegans* may respond differently to a microbiome member diet, due in part to worm colonization, compared to a standard *E. coli* OP50 diet.

**Fig. 2:**
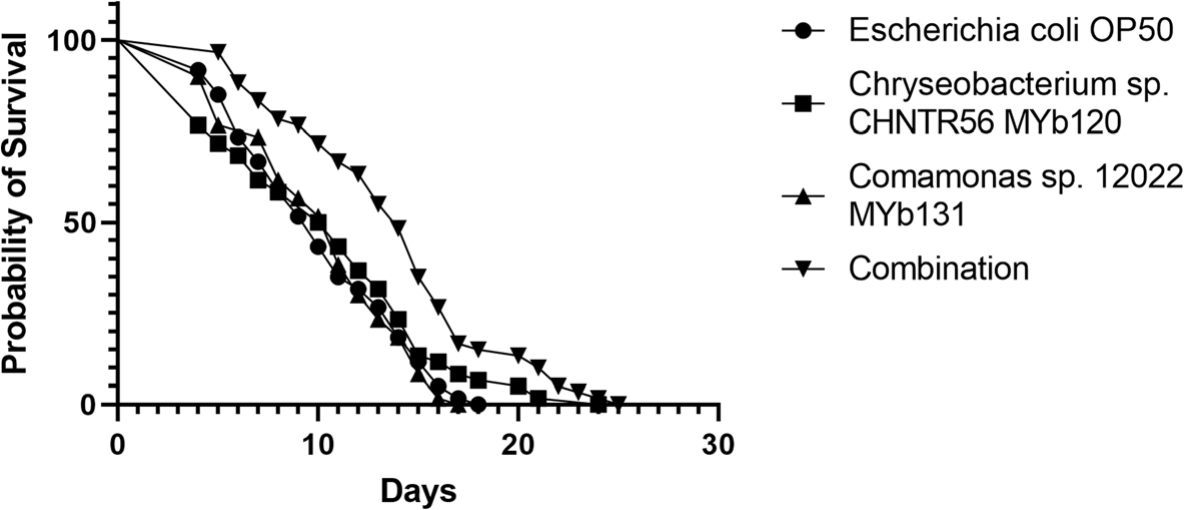
Survival of the worms grown with different bacterial isolates over time. Survival curves are shown. Growth with *Chryseobacterium* sp. CHNTR56 MYb120 alone extended worm maximum lifespan (*P* = 0.0122), and the combination of both native bacteria isolates (*Chryseobacterium sp.* CHNTR56 MYb120 and *Comamonas* sp. 12022 MYb131) increased worm median and maximum lifespans (*P* < 0.0001), compared to the worms grown *E. coli* OP50. This experiment was replicated three times and worms were kept at 21 °C

As the colonizing *Chryseobacterium* sp*.* CHNTR56 MYb120 or *Comamonas* sp. 12022 MYb131 isolates had a benefit on progeny output under stressful conditions, indicating an enhanced cellular protection to toxicity, we questioned whether they could have an influence on worm lifespan. We monitored whether *Chryseobacterium* sp*.* CHNTR56 MYb120 or *Comamonas* sp. 12022 MYb131 when fed to *C. elegans*, influenced worm physiology, resulting in altered lifespan, compared to a standard *E. coli* OP50 diet (Fig. 2, Table 1). The median lifespan of *C. elegans* grown with *E. coli* OP50 was found to be 10, which is close to the range reported for the lifespan of wild type N2 strain under similar conditions where worms are transferred frequently and FUDR is not used [26–29]. The observation of shorter lifespan, in comparison with experiments using FUDR (average mean lifespan can be around 14–16 days) [30, 31], was expected due to longer light exposure (as worms were transferred to fresh plates daily) that causes reduced lifespan in *C. elegans* [32]. In the present study, *C. elegans* grown with *E. coli* OP50 or *Comamonas* sp. 12022 MYb131 showed similar survival rates, however, worms grown with *Chryseobacterium* sp. CHNTR56 MYb120 demonstrated lifespan extension (only maximum, 25% increase, compared to worms grown with *E. coli* OP50, *P* = 0.0122). More interestingly, worms grown with the combination of both native bacterial isolates had an extended overall lifespan (maximum, 41% increase compared to *E. coli* OP50, *P* < 0.0001) and increased median lifespan (40% increase compared to *E. coli* OP50, *P* < 0.0001). Altogether, these data show that growth with *Chryseobacterium* sp. CHNTR56 MYb120 and the combination of bacterial isolates promote *C. elegans* lifespan extension.

**Table 1:** Lifespan of the worms grown with different native bacterial isolates

Bacteria isolate	Number of live worms per replicate	Number of dead worms per replicate	Median survival (days)	Maximum survival (days)	Median lifespan (P value)	Maximum lifespan (P value)
*Escherichia coli* OP50	20	20	10	16	–	–
*Chryseobacterium* sp. CHNTR56 MYb120	20	20	10.5	20	0.3201	0.0122
*Comamonas* sp. 12022 MYb131	20	20	11	16	0.8128	0.5634
Combination	20	20	14	22.5	< 0.0001	< 0.0001

Lifespan values and related statistics for the worms grown with the native bacterial isolates of interest and *E. coli* OP50 are shown. Maximum lifespan of the worms grown with *Chryseobacterium* sp. CHNTR56 MYb120 alone and median and maximum lifespans of the worms grown with the combination of both native bacterial isolates were increased, compared to the worms grown *E. coli* OP50 (*P* = 0.0122 and *P* < 0.0001, respectively). No worms were censored

### The native bacterial isolates upregulate detoxification genes in *C. elegans*

**Fig. 3:**
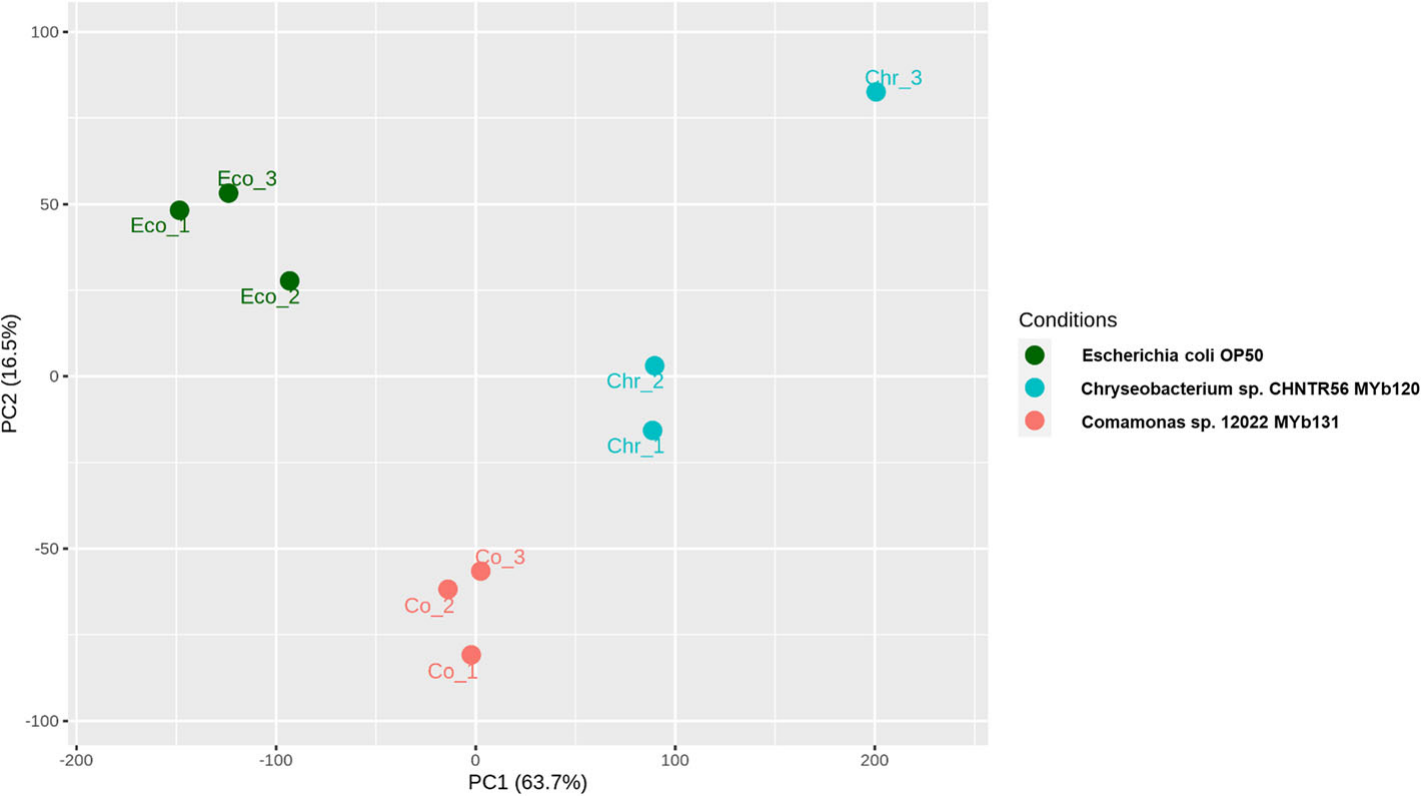
Principal component analysis (PCA). PCA demonstrates the similarity of the samples based on their gene expression patterns in a two dimensional space. These samples include the worms grown with *E. coli* OP50, *Chryseobacterium sp.* CHNTR56 MYb120 or *Comamonas* sp. 12022 MYb131

We further investigated the *C. elegans* transcriptomic response when the worm is grown with members of its microbiome as compared to growth with non-colonizing bacteria, such as *E. coli* OP50. RNAseq analysis of *C. elegans* grown with *Chryseobacterium* sp. CHNTR56 MYb120, *Comamonas* sp. 12022 MYb131 or *E. coli* OP50 yielded around 20 million reads for each sample (*n* = 3, for each phenotype). Approximately 18,000 features were identified and 94% of these features were assigned to the worm’s genes. The similarity of the samples based on their gene expression patterns was inspected by principal component analysis (PCA), which shows a clear separation of the various samples based on the fed diet (Fig. 3). Statistically significant differentially expressed genes (DEGs; defined by edgeR, FDR < 0.05) of the worms grown with the native bacteria (*Chryseobacterium* sp. CHNTR56 MYb120 or *Comamonas* sp. 12022 MYb131) versus *E. coli* OP50 are shown in Additional file 3. The number of DEGs (with fold change greater than 1) for the worms grown with *Chryseobacterium* sp. CHNTR56 MYb120 or *Comamonas* sp. 12022 MYb131, compared to *E. coli* OP50, were 6109 and 3049, respectively, indicating that *C. elegans* is more responsive to *Chryseobacterium* sp. CHNTR56 MYb120. The number of *C. elegans* DEGs induced by each native bacterial isolate is shown in a Venn diagram (Additional file 4, Fig. S2).

**Fig. 4:**
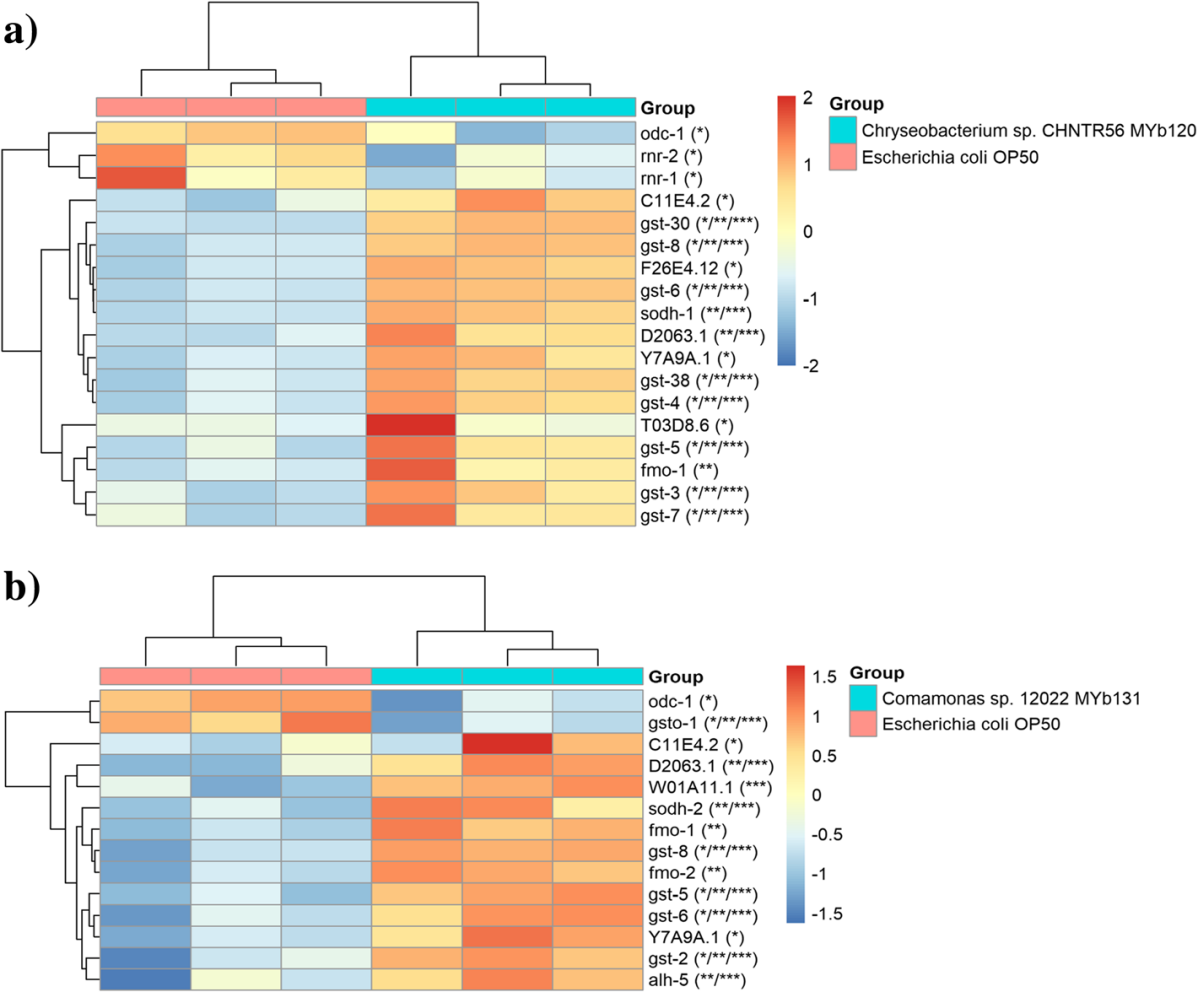
Heatmap for the genes involved in the enriched cellular detoxification related pathways. DEGs were defined by edgeR with FDR < 0.05. Cellular detoxification related enriched genes are shown for worms grown with **a.** *Chryseobacterium* sp. CHNTR56 MYb120 or **b.** *Comamonas* sp. 12022 MYb131. Cellular detoxification related genes were mainly upregulated GSTs. *: Glutathione metabolism (KEGG entry: cel00480). **: Drug metabolism - cytochrome P450 (KEGG entry: cel00982). ***: Metabolism of xenobiotics by cytochrome P450 (KEGG entry: cel00980)

Transcriptome analysis of worms grown with *Chryseobacterium* sp. CHNTR56 MYb120 or *Comamonas* sp. 12022 MYb131 showed enrichment of various biological processes based on gene ontology (GO) annotation (Table 2) and pathways according to KEGG database (Table 3). Most notably, cellular detoxification mechanisms were enriched, which include glutathione metabolism, drug metabolism and metabolism of xenobiotics by cytochrome P450 enzymes in both *Chryseobacterium* sp. CHNTR56 MYb120 and *Comamonas* sp. 12022 MYb131 fed groups, suggesting that the bacterial isolates regulate these biological pathways in the worm. Heatmap analysis revealed DEGs for cellular detoxification mechanisms in worms grown with each bacterial isolate of interest (Fig. 4). These included genes encoding for glutathione S-transferases (GSTs) and other enzymes such as alcohol dehydrogenases (SODH-1 and SODH-2) associated with the translocation of DAF-16, a regulator of longevity, into the nucleus [34–39]. For the *C. elegans* enriched pathways in cellular detoxification (Fig. 4), some genes showed homology with human genes and relation to aging. These included genes that encode protein RNR-2 [*rnr-2*, associated with anti-longevity in *C. elegans* by Human Ageing Genomic Resources (HAGR) [40]; human ortholog: RRM2B, reported by Ortholist2 [41], which was downregulated in *Chryseobacterium* sp. CHNTR56 MYb120 fed group, dimethylaniline monooxygenase [N-oxide-forming] [*fmo-2*, associated with pro-longevity in *C. elegans* by HAGR; human ortholog: FMO3, reported by Ortholist2], which was upregulated in the *Comamonas* sp.12022 fed group, and glutathione S-transferase-5 (*gst-5*, associated with pro-longevity in *C. elegans*; human ortholog: GSTA4, based on Ortholist2), which was upregulated in both groups.

**Table 2:** Top 20 enriched biological processes. Gene enrichment was performed using over-representation analysis with NetworkAnalyst [33]

Pathways	Worms grown with ***Chryseobacterium*** sp. CHNTR56 MYb120	Rank	Hits/Total	P.Value	Worms grown with ***Comamonas*** sp. 12022 MYb131	Rank	Hits/Total	P.Value
*Behaviour*	Locomotory behavior	10	16/40	0.0077	–	–	–	–
*Biological phase*	M phase of mitotic cell cycle	13	4/5	0.00964	–	–	–	–
*Biological regulation*	Regulation of sequence specific DNA binding transcription factor activity	9	10/20	0.00536	Regulation of sequence specific DNA binding transcription factor activity	18	7/20	0.00561
*Cellular component organization or biogenesis*	Protein oligomerization	2	20/39	5.51E-05	Protein oligomerization	19	10/39	0.0118
	Protein homooligomerization	3	19/37	8.26E-05	–		–	–
	Chromosome condensation	7	7/11	0.0035	–		–	–
*Cellular process*	Dephosphorylation	4	48/140	0.00052	Dephosphorylation	2	46/140	1.7E-11
	Protein dephosphorylation	1	48/121	7.6E-06	Protein dephosphorylation	1	46/121	4.1E-14
	Negative regulation of nucleobase containing compound metabolic process	14	25/73	0.0108	Phosphorylation	9	86/504	0.00013
	Neuropeptide signaling pathway	17	9/19	0.0126	Protein phosphorylation	5	86/470	8.7E-06
*Developmental process*	–	–	–	–	Nervous system development	16	50/286	0.00193
	–	–	–	–	Neurogenesis	15	47/262	0.00152
	–	–	–	–	Generation of neurons	14	47/260	0.00129
	–	–	–	–	Neuron differentiation	12	44/230	0.00055
	–	–	–	–	Neuron development	11	41/207	0.0004
	–	–	–	–	Neuron projection development	13	37/192	0.00128
	–	–	–	–	Epithelial cell differentiation	6	13/30	1.2E-05
	–	–	–	–	Epidermis development	20	7/23	0.013
	–	–	–	–	Mesoderm development	17	10/31	0.00195
*Metabolic process*	DNA replication	20	21/62	0.021	–			
	DNA replication initiation	6	5/6	0.00252	Macromolecule modification	4	147/896	3.9E-06
	Negative regulation of RNA metabolic process	11	22/61	0.00838	Protein modification process	3	146/848	2.3E-07
	Negative regulation of transcription, DNA dependent	12	22/61	0.00838	–		–	–
	Negative regulation of transcription from RNA polymerase II promoter	18	16/43	0.0167	–		–	–
*Multicellular organismal process*	Detection of stimulus involved in sensory perception	8	6/9	0.00509	–		–	–
	Regulation of muscle contraction	19	9/20	0.0185	–		–	–
*Multi-organism process*	Spermatid differentiation	16	7/13	0.012	Spermatid differentiation	8	8/13	2.5E-05
	Spermatid development	15	7/13	0.012	Spermatid development	7	8/13	2.5E-05
*Response to stimulus*	Detection of stimulus	5	8/13	0.00238	Response to wounding	10	7/12	0.00013

Enriched biological processes ranked by *P*-value and categorised based on an online resource (Mouse Genome Informatics, https://www.informatics.jax.org/) are shown

**Table 3:** Top 20 enriched biological pathways

Worms grown with ***Chryseobacterium*** sp. CHNTR56 MYb120						Worms grown with ***Comamonas*** sp. 12022 MYb131					
Pathway	Total	Expected	Hits	P.Value	FDR	Pathway	Total	Expected	Hits	P.Value	FDR
DNA replication	33	4.83	17	5.32E-07	6.64E-05	Circadian rhythm - mammal	23	1.43	10	3.49E-07	4.36E-05
TGF-beta signaling pathway	33	4.83	14	9.06E-05	0.00383	TGF-beta signaling pathway	33	2.05	11	2.09E-06	0.000131
Glutathione metabolism*	38	5.56	15	0.000136	0.00383	Drug metabolism - cytochrome P450*	32	1.99	10	1.21E-05	0.000505
Circadian rhythm - mammal	23	3.37	11	0.000144	0.00383	Metabolism of xenobiotics by cytochrome P450*	29	1.81	9	3.63E-05	0.00113
Wnt signaling pathway	64	9.37	21	0.000153	0.00383	Wnt signaling pathway	64	3.98	13	9.65E-05	0.00241
Fatty acid metabolism	56	8.2	17	0.00177	0.0357	Peroxisome	64	3.98	12	0.000403	0.00731
Taurine and hypotaurine metabolism	5	0.732	4	0.002	0.0357	Cysteine and methionine metabolism	31	1.93	8	0.00042	0.00731
Drug metabolism - cytochrome P450*	32	4.69	11	0.004	0.0625	Fatty acid metabolism	56	3.49	11	0.000468	0.00731
Calcium signaling pathway	42	6.15	13	0.00508	0.0706	Glutathione metabolism*	38	2.37	8	0.0018	0.0225
Metabolism of xenobiotics by cytochrome P450*	29	4.25	10	0.00587	0.0734	Taurine and hypotaurine metabolism	5	0.311	3	0.00215	0.0225
Mismatch repair	18	2.64	7	0.01	0.114	Arginine and proline metabolism	39	2.43	8	0.00215	0.0225
Biosynthesis of unsaturated fatty acids	16	2.34	6	0.0206	0.215	Biosynthesis of unsaturated fatty acids	16	0.996	5	0.00216	0.0225
Fatty acid elongation in mitochondria	13	1.9	5	0.0306	0.285	Limonene and pinene degradation	17	1.06	5	0.00291	0.0279
Pyrimidine metabolism	68	9.96	16	0.0319	0.285	Phenylalanine metabolism	18	1.12	5	0.00383	0.0342
Phenylalanine metabolism	18	2.64	6	0.037	0.307	Ubiquitin mediated proteolysis	84	5.23	12	0.00474	0.0395
Neuroactive ligand-receptor interaction	23	3.37	7	0.0404	0.307	Nitrogen metabolism	21	1.31	5	0.00781	0.0611
Cyanoamino acid metabolism	6	0.879	3	0.0442	0.307	Tyrosine metabolism	22	1.37	5	0.00962	0.0707
Sulfur metabolism	6	0.879	3	0.0442	0.307	ECM-receptor interaction	8	0.498	3	0.0105	0.0727
Progesterone-mediated oocyte maturation	39	5.71	10	0.0487	0.315	Lysosome	76	4.73	10	0.0172	0.113
–						alpha-Linolenic acid metabolism	11	0.685	3	0.0269	0.168

Enriched biological pathways (KEGG) ranked by P-value are shown. Cellular detoxification related pathways are enriched in the worms grown with *Chryseobacterium* sp. CHNTR56 MYb120 or *Comamonas* sp. 12022 MYb131, in comparison with *E. coli* OP50. These pathways are indicated with asterisk (*)

The cytochrome P450 family genes (*cyp*) were also upregulated in the worms grown with *Chryseobacterium* sp. CHNTR56 MYb120 (17 out of 23) or *Comamonas* sp. 12022 MYb131 (27 out of 28) (Additional file 5, Fig. S3), which suggests a link between the regulation of CYPs and GSTs. The expression of *cyp* genes along with *gst* genes, is known to be upregulated to reduce toxicity of endogenous and exogenous compounds [42, 43]. In this case, the native bacterial isolates may promote a low level of toxicity to trigger the expression of these enzymes. These genes are probably co-regulated in the worm as GST action follows CYP modification of substrates [44]. Furthermore, a reference gene of oxidative stress, *hsp-16.2* [19], was consequently downregulated, suggesting suppression of oxidative stress, in the worms grown with each bacterial isolate.

**Fig. 5:**
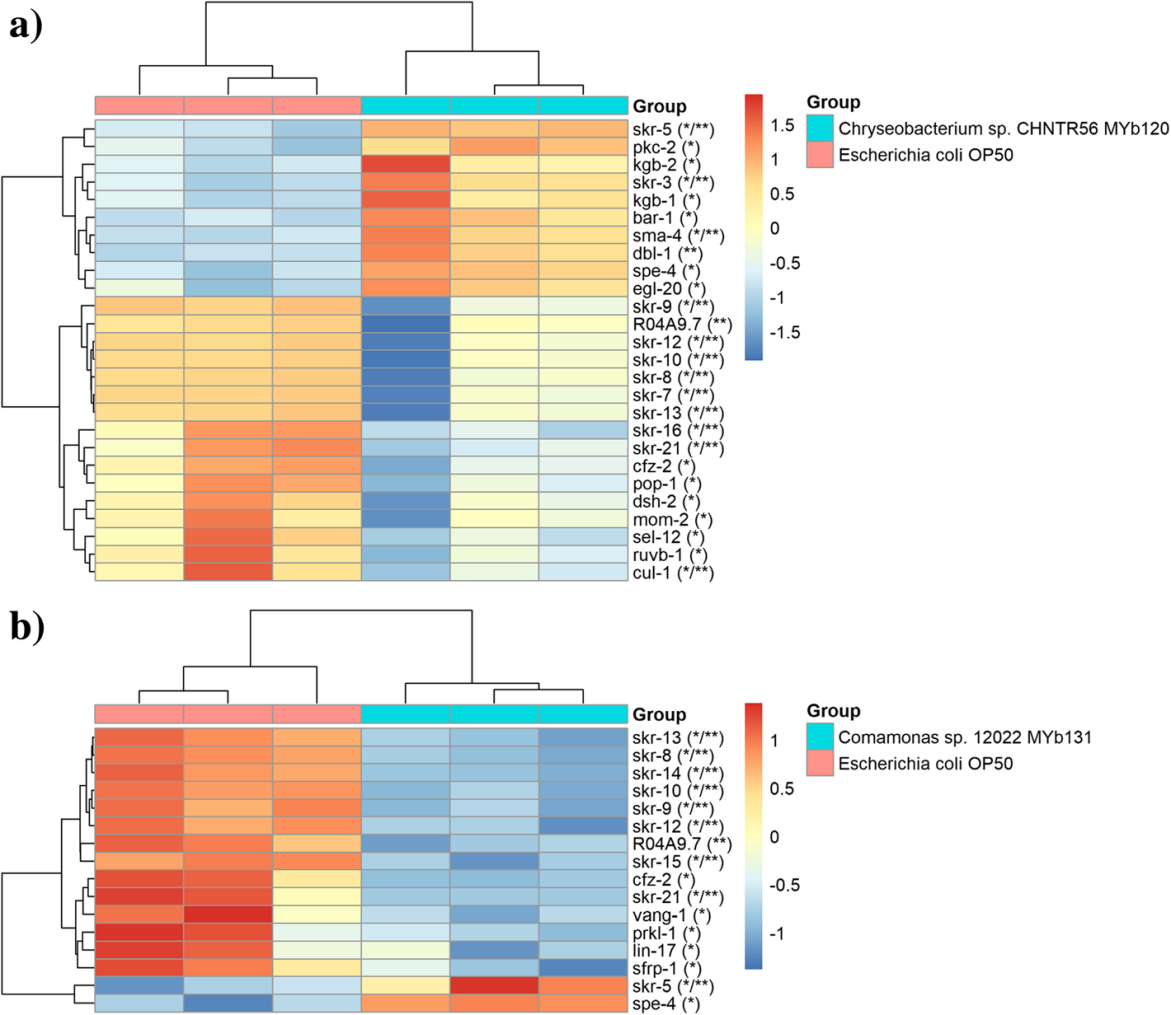
Heatmap for the genes involved in the enriched signaling pathways. DEGs were defined by edgeR with FDR < 0.05. Genes involved in TGF-beta and Wnt signaling were mainly downregulated in the worms grown with both native bacteria, **a.** *Chryseobacterium* sp. CHNTR56 MYb120 or **b. ***Comamonas* sp. 12022 MYb131, in comparison with the worms grown with *E. coli* OP50. *: Wnt signaling. **:TGF-beta signaling

The observed enrichment in cellular detoxification was reversely accompanied with the enrichment of TGF-beta and Wnt signaling pathways, involved in many aspects from body growth, innate immunity to longevity in *C. elegans* [45] (Fig. 5). This reverse relationship (upregulation of cellular detoxification genes but downregulation of the signaling-related genes) reflected the findings reported in long-lived mutants (grown with *E. coli* OP50) [46], citing the effect of the microbiome members on worm longevity. The signaling-related genes were mainly downregulated in both pathways for the worms grown with *Chryseobacterium* sp. CHNTR56 MYb120 or *Comamonas* sp.12022. The *skr* genes were mostly downregulated, except *skr-3* and *skr-5*, in the *Chryseobacterium* sp. CHNTR56 MYb120 group. The Skp1 related (ubiquitin ligase complex component) gene, *skr-5*, having a human ortholog (*SKP1*; Ortholist2), is associated with anti-longevity in *C. elegans* (HAGR) yet the regulation of *skr-5* is indeed conditional [47]. Additionally, beta-catenin/armadillo-related protein 1, having a human ortholog (*CTNNB1*; Ortholist2), is associated with Wnt signaling and pro-longevity in *C. elegans* (HAGR), was upregulated in the *Chryseobacterium* sp. CHNTR56 MYb120 group and protein vang-1, having a human ortholog (VANGL1; Ortholist2), is associated with Wnt signaling and anti-longevity in *C. elegans* (HAGR), was downregulated in the *Comamonas* sp. 12022 MYb131 group. Altogether, these data showed that the transcriptomic response to each of the microbiome members, *Chryseobacterium* sp. CHNTR56 MYb120 and *Comamonas* sp. 12022 MYb131, are different compared to a non-native bacterium, *E. coli* OP50. Both bacterial isolates upregulated the host *C. elegans* genes encoding GSTs, CYPs and downregulated genes in the TGF-beta and Wnt signaling pathways. These responses are suggested to be responsible for the benefit that *Chryseobacterium* sp. CHNTR56 MYb120 and *Comamonas* sp. 12022 MYb131 provide *C. elegans* under stress conditions and could explain the longevity-promoting effect of these bacteria.

### The native bacterial isolates possess genomes containing multiple gene homologs involved in vitamin B6 synthesis

The native bacterial isolates caused regulation of genes associated with synthesis of cysteine (required for glutathione metabolism); *cysl-1, − 2, − 4* were upregulated by *Chryseobacterium* sp. CHNTR56 MYb120 and *cysl-2, − 3, − 4* were upregulated by *Comamonas* sp. 12022 MYb131. This suggests that cysteine synthesis appeared to be favored in *C. elegans* grown with native bacterial isolates, resulting in enhanced worm fitness and lifespan, most probably through supplementation of glutathione levels required for glutathione-mediated detoxification mechanisms (i.e., GSTs). Cysteine synthesis requires the active form of vitamin B6 [pyridoxal 5′-phosphate (PLP); co-factor of CYSL enzymes], based on the information from UniProt (www.uniprot.org) [48], we sought to determine whether the bacterial isolates of interest have the genetic repertoire to provide this vitamin to the worm.

To investigate the potential interdependency between host cysteine synthesis (catalyzed by CYSL enzymes) and vitamin B6 synthesis (co-factor of CYSL enzymes) by the native bacterial isolates, we analyzed the genomes of the bacterial isolates of interest by nanopore sequencing to determine whether the bacteria have genes to encode enzymes that synthesize the active form of vitamin B6. The vitamin B6 de novo and salvage pathways contain many enzymes, as observed in Table 4. The N50 length of the obtained sequences was ~ 8 kb and the assembly process produced one contig (with coverage >89X) for each bacterium. Further details about the size of the assembled genomes with coverage depth, coding sequences and identified genes are shown in Additional file 6.

**Table 4:** Genes that are involved in vitamin B6 metabolism and present in the bacteria genomes

***Chryseobacterium*** sp. CHNTR56 MYb120	***Sphingobacterium faecium*** MYb181	***Comamonas sp.*** 12022 MYb131	***Achromobacter*** sp. F32 MYb9	***E. coli*** OP50
*dxs*	*dxs_1*	*dxs_1*	*dxs_1*	*dxs_1*
*_*	*dxs_2*	*dxs_2*	*dxs_2*	*dxs_2*
				*dxs_3*
*pdxJ_1*	*pdxJ*	*pdxJ*	*pdxJ*	*pdxJ*
*pdxJ_2*	*_*	*_*	*_*	*_*
*pdxH*	*pdxH*	*pdxH*	*pdxH*	*pdxH*
*pdxB_1*	*_*	*pdxB*	*_*	*pdxB*
*pdxB_2*	*_*	*_*	*_*	*_*
***serC_1***	*serC*	*serC*	*serC*	*serC_1*
***serC_2***	*_*	*_*	*_*	*serC_2*
***serC_3***	*_*	*_*	*_*	*_*
***serC_4***	*_*	*_*	*_*	*_*
*pdxA_1*	*pdxA2*	***pdxA2_1***	*pdxA2_1*	*pdxA*
*pdxA_2*	*_*	***pdxA2_2***	*pdxA2_2*	*_*
*_*	*_*	***pdxA2_3***	*pdxA2_3*	*_*
*gapA*	*gapA_1*	*gapA*	*_*	*gapA_1, epd_1*
*_*	*gapA_2*	*_*	*_*	*gapA_2, epd_2*
*_*	*_*	*pdxKǂ*	*pdxKǂ*	*pdxKǂ*
*_*	*_*	*pdxYǂ*	*_*	*pdxYǂ*

Multiple homologs in the specific route of de novo vitamin B6 pathway (D-erythrose 4-P is converted to 3-amino-1-hydroxyacetone 1-P4) for *Chryseobacterium* sp. CHNTR56 MYb120 (serC_1–4) and *Comamonas* sp. 12022 MYb131 (pdxA2_1–3) are present, which are indicated with bold. ǂ: Salvage pathway

Analysis of the bacterial genomes demonstrated that *Chryseobacterium* sp. CHNTR56 MYb120 and *Comamonas* sp. 12022 MYb131 genomes possess various gene homologs for the specific route of the vitamin B6 (pyridoxal 5′-phosphate) de novo synthesis pathway (D-erythrose 4-P is converted to 3-amino-1-hydroxyacetone 1-P4), with the predicted catalytic activity in protein translation [two *pdxB* homologs and four *serC* homologs in *Chryseobacterium* sp. CHNTR56 MYb120 and three *pdxA2* homologs in *Comamonas* sp. 12022 MYb131], in comparison to *E. coli* OP50 (Table 4). The presence of these gene homologs suggests 3-amino-1-hydroxyacetone 1-P4 combines preferentially with D-xylulose 5-P more for vitamin B6 synthesis in these bacteria [as D-xylulose 5-P is a precursor which can be converted to other metabolites [49]]. This is further promoted in *Chryseobacterium* sp. CHNTR56 MYb120 based on the presence of two *pdxJ* homologs. Many genes (including *serC*, *pdxJ*, *pdxB* and *pdxA*) in *Chryseobacterium* sp. CHNTR56 MYb120 were not detected in the closely related species, *Sphingobacterium faecium* MYb181. Multiple *pdxA* homologs, as detected in *Comamonas* sp. 12022 MYb131, are present in the closely related species, *Achromobacter* sp. F32 MYb9. However, *Achromobacter* sp. F32 MYb9 lacks the *gapA*, *epd* and *pdxB* genes, which could translate to significantly less influence of the vitamin B6 de novo synthesis pathway in this bacterium, compared to *Comamonas* sp. 12022 MYb131.

**Fig. 6:**
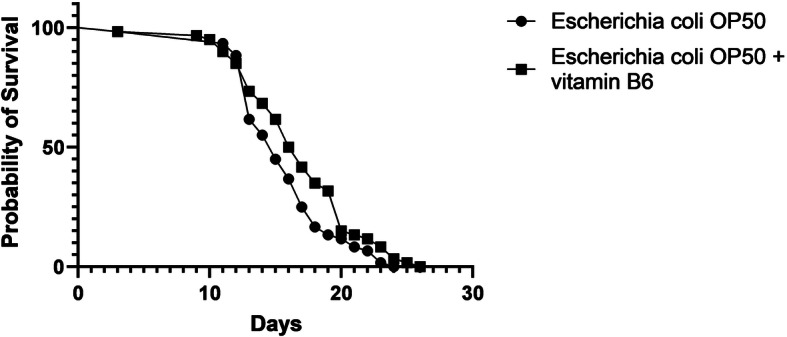
Survival of the worms grown with *E. coli* OP50 in the presence or absence of vitamin B6 supplementation over time. Survival curves are shown. Worms grown with *E. coli* OP50 supplemented with vitamin B6 demonstrated significantly higher median and maximum lifespans, compared to control (*P* = 0.0382 for median lifespan and *P* = 0.0402 for maximum lifespan). The concentration of vitamin B6 (pyridoxine hydrochloride) was 100 nM and this experiment was replicated three times

Based on these observations, we examined the effect of vitamin B6 on worm survival in vivo. Worms grown with the presence of *E. coli* OP50 and vitamin B6 demonstrated significantly higher survival rates (increases from 27 to 58%) after the midlife stage of the worm, compared to control (Fig. 6). The statistics are shown in Additional file 7. The difference in median and maximum lifespans between these groups was found to be statistically significant (*P* = 0.0382 for median lifespan, *P* = 0.0402 for maximum lifespan) (Additional file 7). Consequently, constant supplementation of vitamin B6 resulted in a positive effect on worm lifespan. Altogether, these data support that the presence of the native bacterial isolates of interest is likely favored in the worm in order to achieve stable vitamin B6 supply.

## Discussion

In this study, we first exposed *C. elegans* to certain chemicals (SiO_2_ nanoparticles and juglone) known to cause cellular toxicity through increased oxidative stress by ROS [19, 20] and monitored whether colonizing members of the microbiome could offer a beneficial effect to the worm under the stress conditions. Some bacterial isolates indeed provided a beneficial effect as measured by progeny output of the worms in the presence of SiO_2_ nanoparticles and juglone. Reproductive capacity, based on progeny output, is a measure of *C. elegans* fitness [50]. In our assay, *Chryseobacterium* sp. CHNTR56 MYb120 and *Comamonas* sp. 12022 MYb131 were the only bacterial isolates to significantly promote progeny output under the indicated conditions, which suggests that these bacterial isolates provided a benefit to the worm, more precisely against oxidative stress. This observation highlights the positive influence of the microbiome members on worm reproduction (measured by progeny output), as the response to oxidative stress is generally correlated to reduced capacity for reproduction in higher eukaryotes and *C. elegans* [18, 21, 51].

After confirming that the two bacterial isolates of interest indeed colonized the worm, we then investigated whether they could influence worm lifespan. The lifespan of *C. elegans* grown with *Comamonas* sp. 12022 MYb131 (a native bacterial isolate of the wild isolates of *C. elegans*) and *E. coli* OP50 was not found to be statistically different in the present study, although *Comamonas* DA1877 (relates to *Comamonas aquatica*) was reported to reduce *C. elegans* lifespan (about 2–3 days in mean lifespan) compared to the same control bacteria in previous studies [31, 52]. This could be related to differences of the *Comamonas* isolates, such as secretion and/or colonization properties, which appear to play an important role. Different bacterial strains of the same genus vary in their functional effects on their host as described by previous studies [17, 53]. *C. elegans* grown with *Chryseobacterium* sp. CHNTR56 MYb120 or with a combination of both *Chryseobacterium* sp. CHNTR56 MYb120 and *Comamonas* sp. 12022 MYb131 showed greater survival rates, resulting in an extended lifespan (as maximum lifespan increase by *Chryseobacterium* sp. CHNTR56 MYb120 and as median and maximum lifespan increases by the combination of the two bacteria isolates), compared to growth with *E. coli* OP50 alone (Fig. 2, Table 1), highlighting the effects of the microbiome members on worm longevity. Associated upregulation of *gst* and *cyp* genes, indicating enhanced cellular detoxification, and downregulation of *pdk-1* (which leads to less phosphorylation of SGK-1) (Additional file 3), are proposed to explain the increased ratio of progeny output under oxidative stress and the extension of lifespan, most likely through DAF-16 activity (in the absence of experimentally induced toxicity) [54]. Mechanistically, we suggest that the bacteria can trigger the worm’s cellular detoxification mechanisms through the release of certain molecules (including ROS) that activate the worm response to induce *gst* and *cyp* gene expression. Nevertheless, the downregulation of the *hsp-16.2* gene (a reference gene of oxidative stress [19]) in this study indicates that oxidative stress is eventually suppressed under our experimental conditions.

Although both native bacterial isolates, *Chryseobacterium* sp. CHNTR56 MYb120 or *Comamonas* sp. 12022 MYb131, had similar effects on the worm in terms of progeny production under toxicity and transcriptomic changes in the indicated biological pathway activities (Figs. 4 and 5), their individual effect on worm lifespan was not the same. It is possible that the other factors are involved in the observed results obtained from the lifespan assay. These factors could be of technical nature. For example, we measured progeny production in the presence of toxicity under different experimental conditions compared to the lifespan assay. Nevertheless, the observed differences do not change the main conclusion of our study, as highlighted by the synergism of bacterial isolates resulting in worm lifespan extension.

The detected effects in dealing with toxicity in the worms grown with *Chryseobacterium* sp. CHNTR56 MYb120 or *Comamonas* sp. 12022 MYb131, but not by the other native bacterial isolates including members from the same phyla of these isolates (Additional file 1), and the upregulation of cellular detoxification responses in the worms grown with the two bacterial isolates, suggest that a bacteria buffering effect in dealing with toxicity was not the reason for our observations, given a recent study using a non-colonizing bacteria such as *E. coli* isolates [55], which shows this effect.

**Fig. 7:**
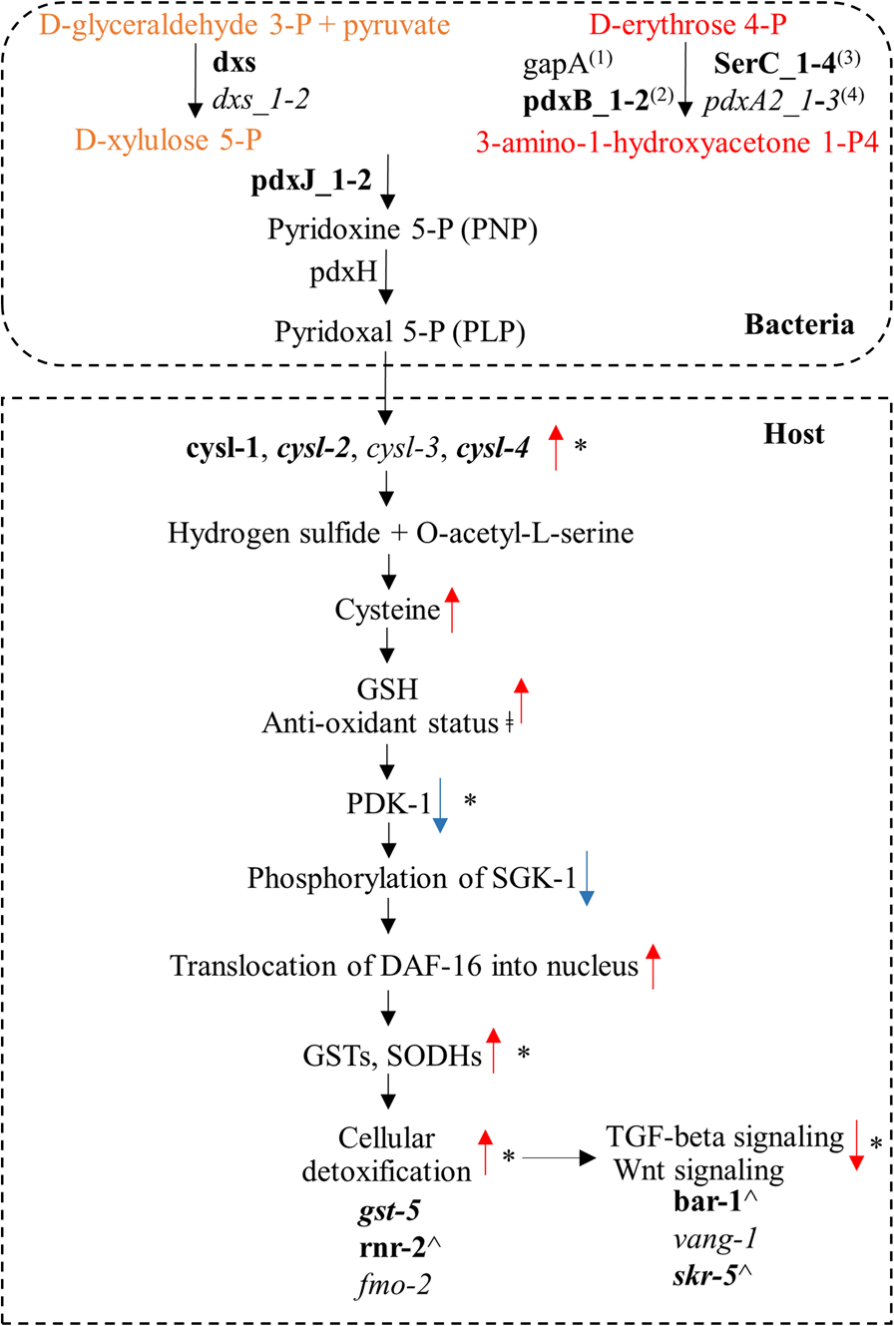
Proposed model of interactions between the worm and its associated microbiome. The active form of bacterial vitamin B6, pyridoxal-5P (PLP), is a required cofactor of cysteine synthases (cysl) of the worm for the enzyme activity. Identification of multiple homologs (serC_1–4 and pdxA2_1–3) in the specific route of vitamin B6 de novo pathway (conversion of D-erythrose 4-P to 3-amino-1-hydroxyacetone 1-P4) in the native bacterial isolates, as determined by nanopore sequencing, and identification of multiple isomers (cysl-1-4) for cysteine synthesis in the host, as determined by RNAseq, demonstrate the critical importance of the interspecies relationship. In this proposed model, vitamin B6 is linked to cellular detoxication through GSTs, which is accompanied with the regulation of TGF-beta and Wnt signaling pathways, where *C. elegans* genes, with human homologs, are regulated. Bacterial genes are highlighted with bold and italic to indicate the relation to *Chryseobacterium* sp. CHNTR56 MYb120 and *Comamonas* sp. 12022 MYb131, respectively. The sequential order for the enzymatic steps is indicated with super script in parenthesis. Red arrow indicates up regulation and blue arrow indicates down-regulation. *: confirmed by RNAseq. ^: reverse gene expression (compared to the other enriched genes in the indicated pathway). ǂ: supported by the downregulation of *hsp-16.2* (detected by RNAseq)

Based on the results shown in the present study, we attributed the observation of the enhanced cellular detoxification to the upregulation of *C. elegans cysl* genes when grown with the isolates (Fig. 7). As cysteine is required for glutathione-mediated cellular detoxification [56–58], we suggest that this is promoted, at least in part, by the bacterial isolates favoring vitamin B6 synthesis, required for cysteine synthesis, as determined by the presence of gene homologs found in their respective genomes. Identification of the multiple *serC* or *pdxA2* homologs in the specific route of vitamin B6 de novo synthesis (D-erythrose 4-P is converted to 3-amino-1-hydroxyacetone 1-P4) suggests that *Chryseobacterium* sp. CHNTR56 MYb120 and *Comamonas* sp. 12022 MYb131 are specialized in the synthesis and expression of vitamin B6, compared to closely related species (analyzed in this study) and *E. coli* OP50.

The increase in the number of gene homologs related to vitamin B6 synthesis in these bacteria is likely related to adaption to diverse and harsh environmental conditions, in which they may encounter oxidative stress [59]. Specifically, the presence of multiple *pdxB* or *pdxJ* homologs in *Chryseobacterium* sp. CHNTR56 MYb120 indicates that the vitamin B6 metabolism is highly critical in this bacterium and is suggested to help the worm to cope with oxidative stress, compared to worms grown with *Comamonas* sp. 12022 MYb131 or *E. coli* OP50. Additionally, available literature also supports our claim that *E. coli* expresses less vitamin B6 compared to soil bacteria [60]. Based on these findings, the colonization effect of the native bacterial isolates was expected to promote stability in vitamin B6 supply at required levels for the worm. Consequently, constant supplementation of NGM plates with vitamin B6 increased worm survival rates (Fig. 6, Additional file 7), thereby further supporting the role of a stable supply of this vitamin in worm physiology. In our colonization experiments, we observed that *E. coli* OP50 is readily consumed by the worm (within about 2 h), therefore it is expected that the worm is not exposed to a constant supply of vitamin B6. Our analysis indicates that supplementing vitamin B6 to the *E. coli* OP50 seeded NGM plates on a regular basis appears to mimic the effect of the colonizing native bacterial isolates of interest (having genomes that contain multiple gene homologs in vitamin B6 synthesis), based on the findings of our lifespan experiments. Additionally, this also indicates that the possible consumption of vitamin B6 by *E. coli* OP50 did not mask the effect of this essential vitamin on the worm.

In the present study, worms grown with *Chryseobacterium* sp. CHNTR56 MYb120 or *Comamonas* sp. 12022 MYb131 showed similar patterns of changes in biological pathway activities at the global level, but differential expression of certain genes associated with aging in these pathways (*rnr-2*, *fmo-2*, *bar-1*, *vang-1*, *ragc-1*) was found to be specific in the response to each bacterial isolate. This observation may explain the synergistic effect of these two bacterial isolates in terms of promoted longevity of the worm. Conversely, a common gene involved in glutathione-based cellular detoxification mechanisms, *gst-5* (related to aging in *C. elegans* and humans), was found to be upregulated in the worms grown with each of the bacterial isolates of interest.

Understanding the effects of the native bacteria on the *C. elegans* host is a very new research field and we aimed to study this complex field by initiating a broad survey of phenotypes followed by omics analyses. The model we use here is established for wild type *C. elegans.* *C. elegans* mutants could not be utilized in recent studies (in terms of focusing on host-microbiota interactions based on native bacterial isolates and their host) [17, 50]. Future studies to establish *C. elegans *mutants harboring the native bacteria will be very useful. Additionally, native bacterial isolates of *C. elegans* have been suggested to release small compounds that are vital for the worm host [61]. Therefore, future studies investigating the bacterially produced metabolites would provide valuable information.

## Conclusions

The present study has shown that two bacterial isolates (*Chryseobacterium* sp. CHNTR56 MYb120 and *Comamonas* sp. 12022 MYb131) of the native *C. elegans* microbiome promote worm fitness (as measured by reproductive capacity in the presence of cellular toxicity caused by SiO_2_ nanoparticles and juglone) and longevity (as illustrated by the synergistic effect of the bacterial isolates). RNAseq and nanopore sequencing suggest that the observed beneficial effect and longevity promotion can be explained, at least in part, by the induction of glutathione-associated cellular detoxification with the upregulation of *cysl* genes in the worm that favors the colonizing native bacterial isolates with diversity in vitamin B6 de novo synthesis, indicating the importance of vitamin supply. This was confirmed by enhanced survival of *C. elegans*, especially in the mid and later stages of life, when co-cultured with vitamin B6. In agreement with other studies [12, 13, 17, 61], this study showcases *C. elegans* as a promising alternative model to study host-microbiome interactions and we expand on this by integrating omics-based analyses.

## Methods

### Study design

L4-young adult *C. elegans*, grown with each of the native bacteria species or *E. coli* OP50, were exposed to chemically-induced toxicities. The progeny produced by the worm were used as the indicator of their fitness or resistance under the challenge. The rest of the experiments were performed independently without exposing the worms to toxicity. The effect of these bacteria on the worm’s lifespan was examined. L4-young adult worms grown with the bacteria of interest were subjected to RNAseq, followed by nanopore sequencing of the genomes of these bacteria. Finally, the effect of the metabolite of interest (vitamin B6) was tested on worm lifespan.

### *Caenorhabditis elegans* and bacteria species

The *C. elegans* N2 strain (Bristol) and *Escherichia coli* OP50 were obtained from the Caenorhabditis Genetics Center (CGC) at the University of Minnesota. A total of 16 native different bacterial isolates (listed in Additional file 1) were kindly provided by Prof. Hinrich Schulenburg, Zoological Institute, Evolutionary Ecology and Genetics, Christian-Albrechts-Universität zu Kiel, Kiel, Germany.

### Worm maintenance and bacterial culture

*Escherichia coli* OP50 and the native bacterial isolates were grown until the bacterial growth reached stationary phase at 37 °C and at 28 °C in tryptic soy broth (TSB), respectively. The *C. elegans* N2 strain was maintained at 21 °C in an incubator on nematode growth media (NGM) plates using *E. coli* OP50. The nematode worms were synchronized using 5.0 ml of alkaline bleach (16.6%, v/v) to kill the adults and to obtain eggs, without any bacterial contamination. Eggs from the adult worms were then washed three times with sterile M9 buffer and left overnight on a rocking platform at room temperature for hatching.

### Colonization assay

Bacterial colonization inside the worm was evaluated based on a previously published method [25]. Firstly, a trial was carried out to determine the antibiotic susceptibility of the bacterial isolates (*n* = 16) by streaking each bacterial isolate on each TSB plate containing various antibiotics such as ampicillin (100 μg/ml), kanamycin (50 μg/ml), tetracycline (10 μg/ml), or mixtures (Additional file 1). Approximately 200 synchronized L1 worms were grown for 48 h at 21 °C to the L4-young adult worm stage with native bacterial isolates (except *Ochrobactrum* sp. R-26465 MYb71 and *Stenotrophomonas* sp. R-41388 MYb57, *Achromobacter* sp. F32 MYb9 because of resistance to all the antibiotic treatments in this study), at equal concentrations of bacteria based on OD_595_ = ~ 5, on NGM plates. The grown worms (*n* = 20) were then transferred to bacteria-free NGM plates including the indicated antibiotics, of which, bacterial isolates are susceptible (Additional file 1) for 24 h for surface sterilization of the worms. The worms were washed with M9 buffer five times, disrupted and placed on TSB agar plates to recover bacteria.

### Progeny assay under chemically-induced toxicity

Approximately 200 synchronized L1 of *C. elegans* were grown to the L4-young adult stage on NGM plates seeded with *E. coli* OP50 or one of the native bacterial isolates (*n* = 16) (at equal concentrations of bacteria based on OD_595_ = ~ 5). The worms were visually inspected as being L4-young adults before being run through the experiment. Five L4-young adult hermaphrodites were transferred to an individual well in quadruplicate of a 12-well plate containing S-medium [62] supplemented with either 0 (control, only S-medium) or 50 μg/ml of silicon dioxide nanoparticles (SiO_2_) [18] and *E. coli* OP50 or one of the native bacteria species at a final concentration of OD_595_ = ~ 1. The L4-young adults were then incubated for 96 h at 21 °C, and resulting larvae were counted by dilution. The ratio of progeny was determined based on the percentage of larvae number in the SiO_2_ treatment to larvae number in control for the worms grown with each tested bacterium. For the phenotypes showing a higher ratio of progeny (> 50% of the control value), the same protocol was followed by using SiO_2_ at the indicated concentration or 50 μM of juglone (5-hydroxy-1,4-naphthoquinone, prepared in ethanol), which was repeated in triplicate. In both initial and final screenings, the progeny of output for *E. coli* OP50 in each well of the plate was measured. We chose SiO_2_ and juglone as both chemicals have been shown to mediate their toxic effects by causing cellular oxidative stress and suppress detoxification mechanisms involving the expression/activity of GSTs [19, 20, 23, 63].

### Lifespan analysis

Approximately 200 synchronized L1 of *C. elegans* were grown to the L4-young adult stage on NGM plates seeded with *E. coli* OP50 or the bacteria of interest (*Chryseobacterium* sp. CHNTR56 MYb120 or *Comamonas* sp. 12022 MYb131) (at equal concentrations of bacteria based on OD_595_ = ~ 5). Worms (*n* = 20 for each group) were transferred to new NGM plates seeded with the corresponding bacteria each day using a sterile platinum wire and alive worms were counted based on motility (death of worms was verified by probing with a platinum wire) [64]. To understand the effect of vitamin B6 in lifespan, a similar protocol was followed with slight modifications; *E. coli* OP50 was the bacterial food source and NGM plates contained 25 μM 5-Fluoro-2′-deoxyuridine (FUDR was added at the days 2 and 6 to stop progeny) plus 100 nM pyridoxine hydrochloride (media stable version of vitamin B6; prepared in M9 buffer as not to change the pH of the NGM media), and worms (*n* = 20 for each group) were transferred to fresh NGM plates with the supplementation of vitamin B6 or M9 buffer alone (as control) every 2–3 days. *C. elegans* possesses two genes encoding putative pyridoxal kinase (PDXK-1) and putative pyridoxamine 5′-phosphate oxidase (F57B9.1), converting pyridoxine into pyridoxine 5′-phosphate and pyridoxine 5′-phosphate to pyridoxal 5′-phosphate (active form), respectively (UniProt; www.uniprot.org) [48]. Each experiment (measuring the effect of native bacteria or the effect of vitamin B6 on lifespan) was repeated in triplicate.

### Total RNA extraction from *C. elegans* for RNAseq

Approximately 200 synchronized L1 of *C. elegans* were grown to the L4-young adult stage on NGM plates seeded with *E. coli* OP50 or bacterium of interest (*Chryseobacterium* sp. CHNTR56 MYb120 or *Comamonas* sp. 12022 MYb131), at equal concentrations of bacteria based on OD_595_ = ~ 5, at 21 °C, in triplicate. The NGM plates containing L4-young adult stage worms were washed twice with M9 buffer and centrifuged at 1000 g for 2 min. A total of 200 μl of Trizol (Ambion, USA) was added to the worm pellet. The worm pellet was snap-frozen in liquid nitrogen, which was followed by a quick thaw. These two steps were repeated once. Total RNA from the homogenate was extracted using the Direct-zol RNA miniprep kit (Zymo Research, USA) according to the manufacturer’s instructions. Quantity and purity of total RNA were analysed using a spectrophotometer (ND-1000, NanoDrop). The RNA samples were sent to the McGill University and Génome Québec Innovation Centre (https://gqinnovationcenter.com) under recommended conditions for quality analysis of total RNA with Bioanalyzer (Agilent) and single-end read (100 base) next generation sequencing of RNA using HiSeq 2500 (Illumina) was performed.

### RNAseq data analysis

Raw data for all samples were obtained in fastq file format from the McGill University and Génome Québec Innovation Centre. Data analysis was carried out using NetworkAnalyst (https://www.networkanalyst.ca) [33]. Read quality was checked with FASTQC (version 0.72) and adapter related sequences were removed using Trim Galore (version 0.4.3.1) (https://www.bioinformatics.babraham.ac.uk/projects/). The genome sequence of *C. elegans* and GTF file (Caenorhabditis_elegans.WBcel235.94.gtf) was obtained from ENSEMBL (https://www.ensembl.org/). Reads were aligned to the *C. elegans* genome with HISAT2 (Galaxy version 2.1.0) [65] and read counts were obtained using HTSeq (version 0.9.1) [66] with the intersection-strict mode. For downstream analysis, sequence data was uploaded to NetworkAnalyst. Sequences with low variance (15%) and low abundance (*n* < 4) counts were removed. Data were normalized based on trimmed mean of M-values and sample distribution was determined by principal component analysis. Differential gene expression analysis was performed using edgeR [67] and statistical significance was defined by the FDR value less than 0.05. For significant gene analysis, overrepresentation enrichment network and heatmap clustering were performed. Biological processes or pathways were considered significant if their *P* values were less than 0.05 from the enrichment analysis. Heatmap visualization was performed with pheatmap function in R.

### Meta-analysis

All the differentially expressed genes (DEGs, FDR < 0.05) in the worms grown with *Chryseobacterium* sp. CHNTR56 MYb120 or *Comamonas* sp.12022 MYb131 (compared to the worms grown with *E. coli* OP50) were searched against aging associated *C. elegans* and human genes using Human Ageing Genomic Resources (HAGR) (https://genomics.senescence.info/) [40]. The aging related genes in the database were defined mainly based on the previous experimental data and biological links (using the indicated data) for *C. elegans* and human, respectively. Moreover, *C. elegans* genes that were associated with aging but not with their human homologs, which were not reported in the database list, were further searched using the data from Ortholist2 [41].

### Genomic DNA extraction of bacteria and nanopore sequencing

*E. coli* OP50, *Chryseobacterium* sp. CHNTR56 MYb120 and *Comamonas* sp. 12022 MYb131 were grown in TSB broth at 28 °C until the growth OD_595_ reached a value of 3. Bacteria were centrifuged at 4000 rpm for 10 min and supernatants were taken off. Genomic DNA from bacteria were extracted using ZymoBIOMICS DNA Miniprep Kit (Zymo Research). DNA quality and quantity were checked with a spectrophotometer (NanoDrop) and Qubit dsDNA BR Assay Kit (Invitrogen), respectively. For comparative purposes, genomic DNA from the other bacteria genetically close to the native bacteria of interest (*Sphingobacterium faecium* MYb181 and *Achromobacter* sp. F32 MYb9; determined based on relevant 16S ribosomal RNA sequences from NCBI using MUSCLE, European Bioinformatics Institute, https://www.ebi.ac.uk) was obtained with the same approach for sequencing. DNA library preparation for each sample including DNA repair, end-prep and adapter ligation steps was carried out using the required reagents (FFPE DNA Repair Mix, Ultra II End Repair/dA-Tailing and Quick Ligation Modules, and Blunt/TA Ligase Master Mix) from New England Biolabs as described in the sequencing protocol provided by Oxford Nanopore Technologies. All DNA clean-up steps were done with Agencourt AMPure XP beads (Beckman Coulter) using a magnetic stand (GE Healthcare). Samples were barcoded with Native Barcoding Expansion kit [EXP-NBD104, Oxford Nanopore Technologies (ONT)]. Pooled DNA of the samples with different barcodes at equal concentrations were prepared for sequencing with Ligation Sequencing Kit (SQK-LSK109, ONT) and loaded onto a flowcell (FLO-MN106, ONT) which is connected to the sequencing device, MinION (ONT), and whole genome long-read sequencing was performed based on the protocol.

### Nanopore sequencing data analysis

After the sequencing run (72 h) was completed with MinKNOW Core (v3.6.5, ONT) on a laptop computer, Fast5 data was converted to Fastq data with Guppy (v3.4.5, ONT). Reads were demultiplexed by qcat (v1.1.0) (https://github.com/nanoporetech/qcat). Quality filtering and read trimming were carried out with Filtlong (v0.2.0) (https://github.com/rrwick/Filtlong) and Porechop (v0.2.4) (https://github.com/rrwick/Porechop), respectively. The obtained sequences were assembled by Flye (v2.7) [68], and coding sequences were determined and annotated by Prokka (v1.14.6) [69]. The outcome of each sequence assembly, genome coverage, coding sequences and gene symbols was reported for each bacterium analyzed. Details in gene functions and enzyme reactions were searched using the data from UniProt (www.uniprot.org) [48]. Genes and metabolites in the vitamin B6 synthesis pathway for bacteria were obtained from previous studies [70, 71].

### Statistical analysis

The group differences for the ratio of progeny output was examined by ANOVA with Tukey’s HSD in R, respectively. For comparison of median lifespan, statistical differences in survival curves were evaluated by log-rank (Mantel-Cox) test using GraphPad Prism (v8.4.3). Comparison of maximum lifespan (defined as 10% of the longest-lived worms) was carried out using Student’s t test [72] with the same software. A *P* value less than 0.05 was accepted as statistically significant.

## Supplementary Information

**Additional file 1: List of native bacterial isolates of** ***C. elegans*** **and susceptibility to antibiotics.** A total of 16 native bacterial isolates, used in this study, is shown with phylum to family information (based on NCBI) and their growth/non-growth status under certain antibiotic treatments.

**Additional file 2: Figure S1. Colonization of** ***C. elegans*** **by the native bacterial isolates of interest.** Bacterial colonies from *C. elegans* grown with *E. coli* OP50 or native bacterial isolates (*Chryseobacterium sp.* CHNTR56 MYb120 and *Comamonas* sp. 12022 MYb131), after surface sterilization of the worms by the indicated antibiotics and without bacterial food source supplementation for 24 h, are shown.

**Additional file 3: Differentially expressed genes between worms grown with different bacterial isolates.** Differentially expressed genes between worms grown with *E. coli* OP50 and *Chryseobacterium sp.* CHNTR56 MYb120 or *Comamonas sp.* 12022 MYb131 were determined by edgeR and ranked based on FDR.

**Additional file 4: Figure S2. Venn diagram.** Statistically significant differentially expressed genes with fold change greater than 1 for *C. elegans* grown with *Chryseobacterium sp.* CHNTR56 MYb120 or *Comamonas sp.* 12022 MYb131, compared to *E. coli* OP50, are shown.

**Additional file 5: Figure S3. Heatmap for the regulation of cytochrome P450 family genes (CYPs).** Most CYPs were upregulated in worms grown with *Chryseobacterium sp.* CHNTR56 MYb120 (a) or *Comamonas* sp. 12022 MYb131 (b), in comparison with *E. coli* OP50.

**Additional file 6: Nanopore sequencing of bacteria genomes.** The number of the contigs, length and annotated genes for each bacterial isolate and *E. coli* OP50 are shown.

**Additional file 7: Lifespan of the worms grown with** ***E. coli*** **OP50 in the presence or absence of vitamin B6.** Lifespan values and related statistics for the worms grown with *E. coli* OP50 alone or *E. coli* OP50 plus vitamin B6 are shown. Median and maximum lifespans of worms grown with *E. coli* OP50 supplemented with vitamin B6 were significantly higher, compared to control (*P* = 0.0382 for median lifespan and *P* = 0.0402 for maximum lifespan). No worms were censored.

## Data Availability

The RNAseq dataset generated and analyzed in this study is available in the NCBI Gene Expression Omnibus (GEO) database repository, with the accession number GSE143794. The nanopore sequencing data is available in the NCBI Sequence Read Archive (SRA), with the accession number PRJNA663603.

## Abbreviations

CGC: Caenorhabditis Genetics Center
CYPs: The cytochrome P450 family genes
DEGs: Differentially expressed genes
FISH: Fluorescence In Situ Hybridization
FUDR: 5-Fluoro-2′-deoxyuridine
GO: Gene ontology
GSTs: Glutathione S-transferases
HAGR: Human Ageing Genomic Resources
KEGG: Kyoto Encyclopedia of Genes and Genomes
NCBI: The National Center for Biotechnology Information
NGM: Nematode Growth Media
PCA: Principal component analysis
RNAseq: RNA sequencing
SiO2: Silicon dioxide
TSB: Tryptic soy broth
UniProt: The Universal Protein Resource
