# Supplementary figures and images for: The symbiotic relationship between Caenorhabditis elegans and members of its microbiome contributes to worm fitness and lifespan extension
Source: BMC Genomics. 2021 May 19;22:364. doi: 10.1186/s12864-021-07695-y (PMC8136213; doi:10.1186/s12864-021-07695-y)

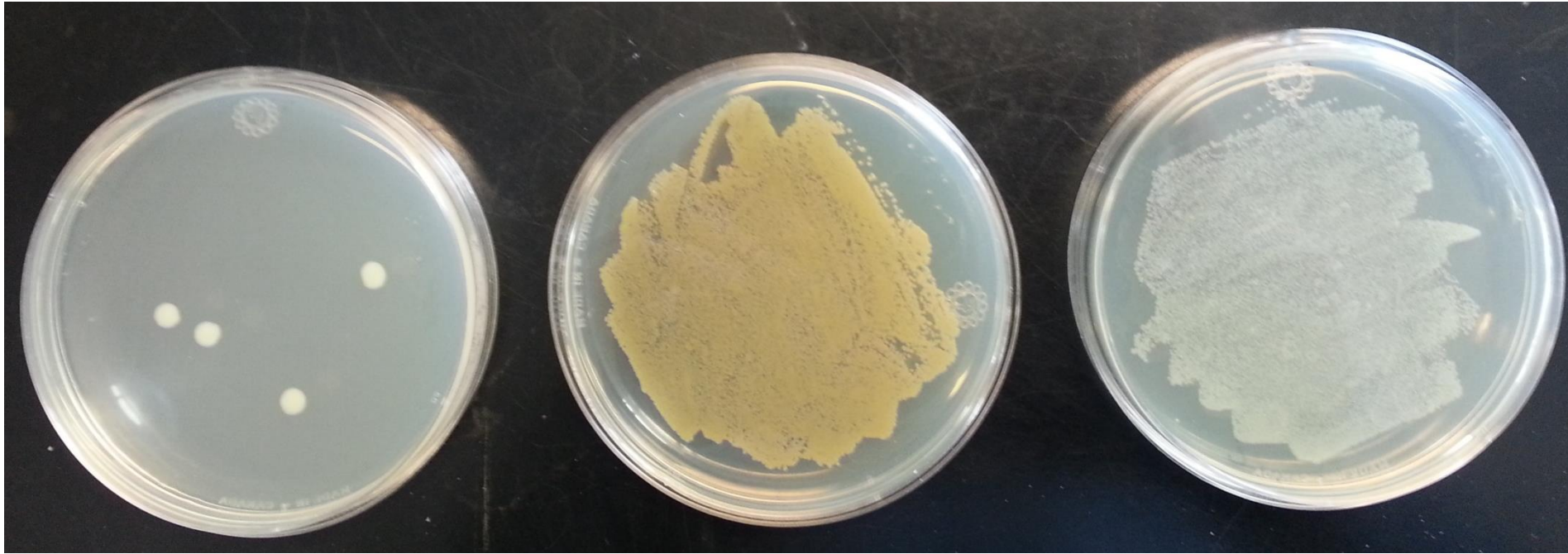

*Escherichia coli* OP50

*Chryseobacterium* sp. CHNTR56 MYb120

*Comamonas* sp. 12022 MYb131

Supplement: Supplementary file 2 — Additional file 2: Figure S1. Colonization of C. elegans by the native bacterial isolates of interest. Bacterial colonies from C. elegans grown with E. coli OP50 or native bacterial isolates (Chryseobacterium sp. CHNTR56 MYb120 and Comamonas sp. 12022 MYb131), after surface sterilization of the worms by the indicated antibiotics and without bacterial food source supplementation for 24 h, are shown. [file 12864_2021_7695_MOESM2_ESM.pdf]

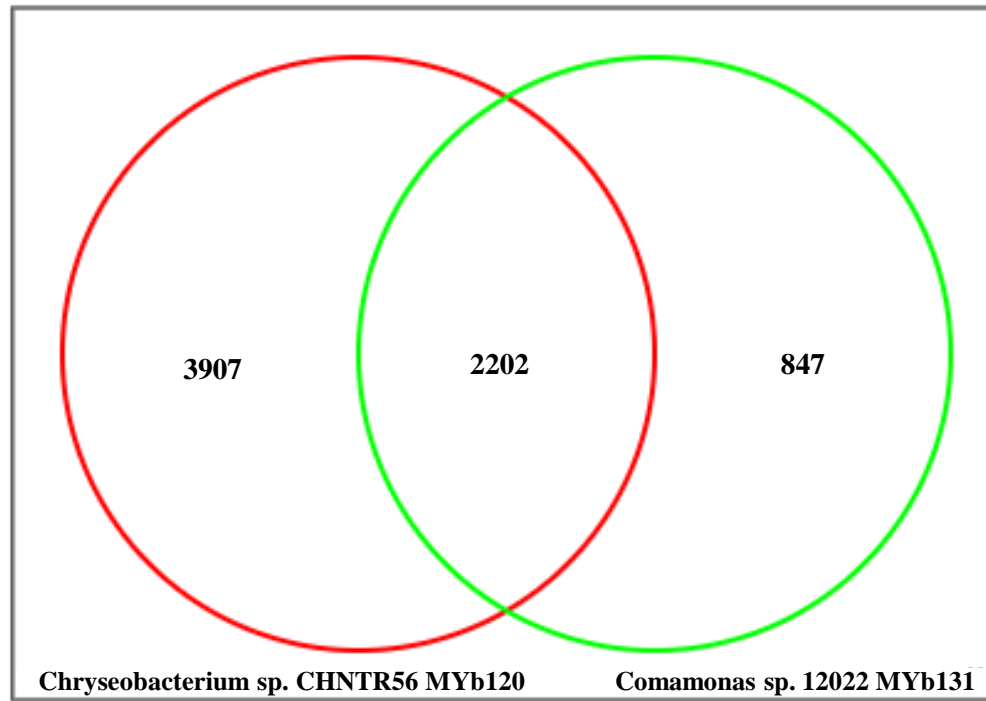

Supplement: Supplementary file 4 — Additional file 4: Figure S2. Venn diagram. Statistically significant differentially expressed genes with fold change greater than 1 for C. elegans grown with Chryseobacterium sp. CHNTR56 MYb120 or Comamonas sp. 12022 MYb131, compared to E. coli OP50, are shown. [file 12864_2021_7695_MOESM4_ESM.pdf]

a)

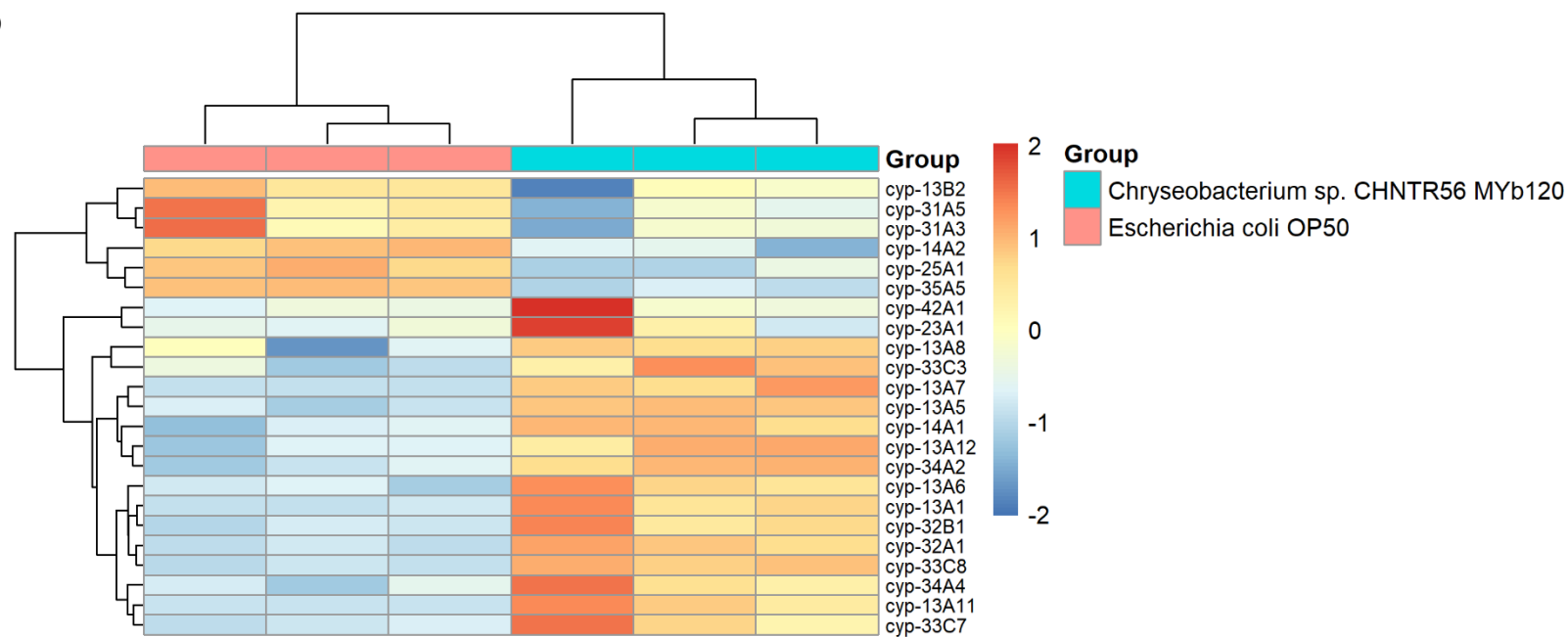

b)

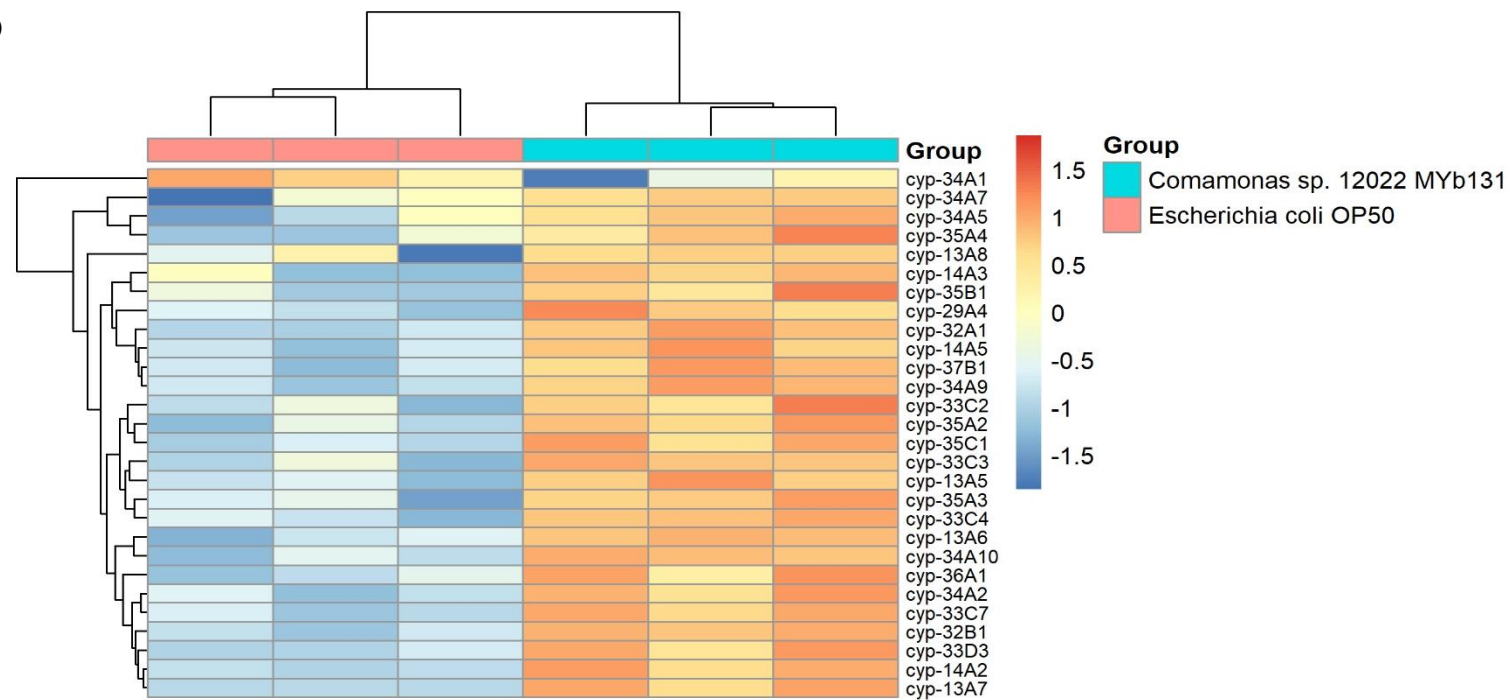

Supplement: Supplementary file 5 — Additional file 5: Figure S3. Heatmap for the regulation of cytochrome P450 family genes (CYPs). Most CYPs were upregulated in worms grown with Chryseobacterium sp. CHNTR56 MYb120 (a) or Comamonas sp. 12022 MYb131 (b), in comparison with E. coli OP50. [file 12864_2021_7695_MOESM5_ESM.pdf]
